# *Mycobacterium wolinskyi* as an emerging cause of pacemaker pocket infection and lead endocarditis: a case report and genomic characterization

**DOI:** 10.1128/asmcr.00146-25

**Published:** 2025-10-21

**Authors:** Julie Dom, Reinoud Cartuyvels, Anne Bruggemans, Timo Froyen, Petra Hilkens, Koen Magerman, Steven Martens, Britta Van Meensel, Jozef Dingemans

**Affiliations:** 1 Clinical laboratory of Microbiology, Jessa Hospital 82256 , Hasselt, Belgium; 2 Department of Infectious Diseases and Immunity, Jessa Hospital 82256 , Hasselt, Belgium; 3 Clinical laboratory of Molecular Microbiology, Jessa Hospital 82256 , Hasselt, Belgium; 4 Department of Immunology and Infection, Hasselt University 54496, Hasselt, Belgium; Rush University Medical Center, Chicago, Illinois, USA

**Keywords:** whole genome sequencing, 16S rRNA sequencing, pacemaker pocket infection, rapidly growing mycobacteria, non-tuberculous mycobacteria, *Mycobacterium wolinskyi*

## Abstract

**Background:**

Rapidly growing non-tuberculous mycobacteria (NTM) are increasingly recognized as causative agents in healthcare-associated infections. *Mycobacterium wolinskyi*, first described in 1999, has been associated with post-traumatic and post-operative wound infections.

**Case Summary:**

We report a case of *M. wolinskyi* pacemaker pocket infection and lead endocarditis in a 73-year-old man, 3 weeks after implantable cardioverter-defibrillator (ICD) implantation. Initial culture from a superficial pocket swab yielded slow-growing, white colonies on chocolate agar, and the strain was identified by MALDI-TOF as *M. wolinskyi*. However, the 16S rRNA, *hsp65*, *rpoB,* and *recA* sequences were identical to those of the unofficially described *M. jacuzzi* species but different from the corresponding sequences of the *M. wolinskyi* ATCC 700010 reference strain, which showed an average nucleotide identity (ANI) of 97.49% upon whole genome comparison. Therapy consisted of ICD removal, surgical debridement of the pocket, and prolonged combination therapy with oral moxifloxacin and co-trimoxazole, with favorable clinical outcome.

**Conclusion:**

This case demonstrates that our strain of *M. wolinskyi* could be an emerging pathogen in device-associated infections. The case highlights the diagnostic limitations of routine methods and emphasizes the role of 16S sequencing and whole genome sequencing in accurate taxonomic classification of NTM species. Broader genomic surveillance is warranted to understand the epidemiology and taxonomy of this rapidly growing NTM.

## INTRODUCTION

*Mycobacterium wolinskyi* is a rapidly growing non-tuberculous mycobacterium first described in 1999 by Brown et al. (1). Although a rare pathogen, this species had been associated with post-operative wound infections and implantable device infections in both immunocompetent and immunocompromised patients (2).

## CASE PRESENTATION

A 73-year-old man with extensive cardiac history presented to the emergency department with suspected pocket infection 3 weeks after implantable cardioverter-defibrillator (ICD) implantation with cardiac resynchronization therapy (CRT-D). Three days after suture removal, swelling and purulent drainage at the surgical site developed. Blood cultures and a superficial pocket swab were obtained, and flucloxacillin 1 g six times daily intravenously was initiated. Transesophageal echocardiography (TEE) revealed vegetations on two pacemaker leads.

Bacterial culture from the superficial pocket swab yielded small, white colonies on chocolate agar (GC-CHOCO, BD) after 5 days of incubation at 35°C with 5% CO_2_. No identification was obtained by MALDI Biotyper Sirius System (Bruker) using MBT Compass HT IV Library (2023). Gram staining of the colonies on GC-CHOCO showed fine Gram-positive rods. Culture on Columbia agar with 5% sheep blood (Col-5%S) and MacConkey agar (MC II) remained negative.

Because of persistent purulent drainage, the ICD was removed. A deep wound swab was taken from the pocket, and both leads were sent to the laboratory. Conventional cultures as well as mycobacterial cultures were performed using Löwenstein–Jensen medium and BD BACTEC MGIT 320 system (BD Diagnostic Systems). Meanwhile, the strain on GC-CHOCO of the superficial swab was identified as *M. wolinskyi* (score 1.83) on MALDI-TOF using the MBT HT Mycobacteria IVD Module (library 2023).

MGIT cultures became positive after 3 days of incubation. Ziehl-Neelsen staining was negative for acid-fast bacteria for all samples, leading to the mycobacterial culture being considered as false positive. Decontamination of original MGIT tubes was performed, and a second MGIT culture was inoculated. However, upon closer examination of Ziehl-Neelsen preparations, Ziehl-negative coccobacilli were observed in all samples, and Auramine stain was positive. Based on the suspicion of mycobacterial infection, MALDI-TOF on MGIT cultures from both leads and the deep swab was performed using MBT HT Mycobacteria IVD Module (library 2023) and yielded *M. wolinskyi* (scores 1.88–1.95). Conventional cultures on Col-5%S, GC-CHOCO, and blood cultures remained negative.

Full-length 16S Nanopore sequencing was performed directly on both pacemaker leads and an isolate obtained from the superficial swab to confirm the unexpected identification of the strain on GC-CHOCO. DNA was extracted from 250 µL Brain Heart Infusion (BHI) broth in which each pacemaker lead was incubated or 250 µL of a 0.5 McFarland solution (from the superficial wound swab isolate) using the ZymoBIOMICS DNA Miniprep Kit (Zymo Research), followed by amplification of the full length 16S rRNA gene via the 16S Barcoding Kit 24 V14 (SQK-16S114.24) from Oxford Nanopore Technologies and sequencing on a MinION R10.4.1 flow cell for a total of 72 h with Super Accuracy Basecalling using MinKnow version 24.06.16. Data analysis using BugSeq and 1928 Diagnostics pipelines revealed discrepant results, BugSeq reported ‘*M. wolinskyi*’ and 1928 Diagnostics *‘M. jacuzzii*’ for both pacemaker lead samples as well as an isolated colony from the superficial wound swab. Notably, these strains were present in significant quantities within the clinical samples (Table 1).

**TABLE 1:** Overview of the microbiological and molecular results per sample

Material	Zieh-Neelsen staining	16S rRNA sequencing result	Estimated load (based on 16S)^*c*^	Culture result	MALDI-TOF ID
Wound swab pocket (superficial)	Not performed	*M. jacuzzii^a^*/*M. wolinskyi^b^*	Not determined	Positive	*M. wolinskyi*
Wound swab pocket (deep)	Negative	Not determined	Not determined	Positive	*M. wolinskyi*
Pacemaker lead 1	Negative	*M. jacuzzii*^*a*^/*M. wolinskyi^b^*	2.01 × 10^7^ CFU/ml	Positive	*M. wolinskyi*
Pacemaker lead 2	Negative	*M. jacuzzii^a^*/*M. wolinskyi^b^*	6.77 × 10^8^ CFU/ml	Positive	*M. wolinskyi*

^*a*^ Result reported by 1928 Diagnostics (16S pipeline version 2025-02.3).

^*b*^ Result reported by BugSeq (version 2025-02-19).

^*c*^ The bacterial load was estimated based on the ZymoBIOMICS Spike-in Control II (low microbial load) that was added to each clinical sample (both pacemaker leads) prior to DNA extraction.

Because of this discrepancy, whole genome sequencing (WGS) was performed. DNA was extracted from a bacterial suspension (prepared via dissolving a 10-µL inoculation loop of a pure culture from the superficial wound swab in 450 µL of DNA/RNA shield) using the ZymoBIOMICS DNA Miniprep Kit (Zymo Research) and sequencing using the Rapid Barcoding Kit 96 V14 (SQK-RBK114.96) according to the Nanopore-only Microbial Isolate Sequencing Solution (NO-MISS) protocol using 200 ng DNA input. Sequencing was performed on a MinION R10.4.1 flow cell for a total of 72 h, followed by Super Accuracy Basecalling using MinKnow version 24.06.16, while genome assembly and annotation was performed using wf-bacterial genomes workflow in EPI2ME v1.4.1 (Oxford Nanopore Technologies) or via BugSeq to perform metagenomic analysis, MLST typing, identification of plasmids and antibiotic resistance genes. WGS in Bugseq revealed that the isolated *Mycobacterium* had a genome size of approximately 7.4 million base pairs, was classified as *M. wolinskyi*, contained two ribosomal RNA operons, harbored no plasmids, and that resistance markers *ampC*, *aph(3”*), *bla*, *cml*, *erm*, *rox*, *tet*, and *tet(V*) were present on the chromosome (GenBank Accession Number CP185960.1).

**Fig 1:**
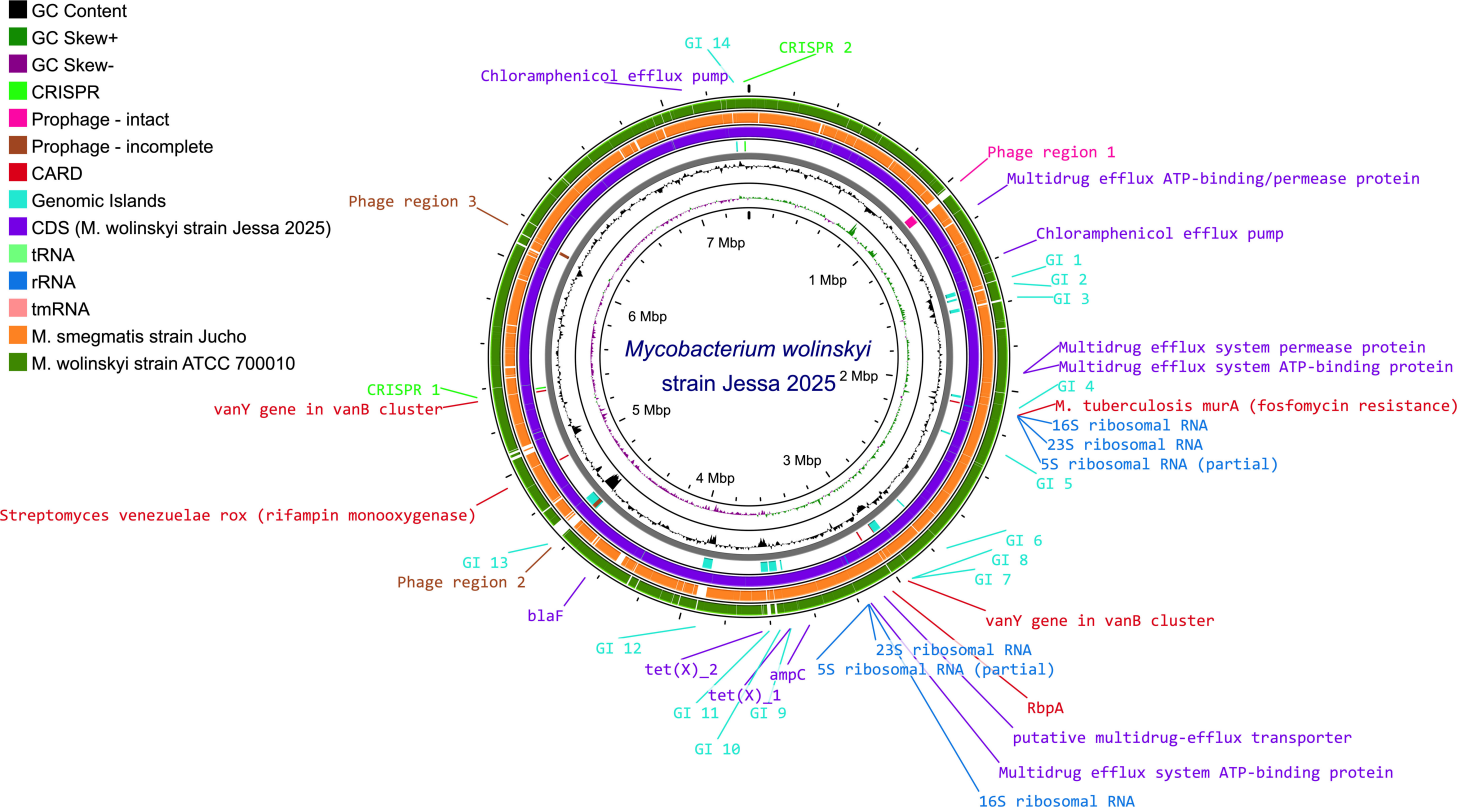
Genome alignment of the strain described in this study (purple) with the *M. smegmatis* (orange) and *M. wolinskyi* (green) reference genomes. Genomic islands (cyan) were predicted using IslandPath-DIMOB prediction method in IslandViewer 4 (7), whereas phage regions (magenta and brown) were predicted using the PHASTEST tool in Proksee. Antibiotic resistance markers predicted by the CARD Resistance Gene Identifier are indicated as well as efflux pumps are highlighted in red and purple, respectively. All features, as well as the complete image, were generated using Proksee. The average nucleotide identity (ANI) compared with *M. smegmatis* strain Jucho and *M. wolisnkyi* ATCC 700010 was 82.62% and 97.49%, respectively.

Further alignment of the genome of the mycobacterial isolate with reference genomes of *M. smegmatis* (strain Jucho) and *M. wolinskyi* (strain ATCC 700010) using Proksee (3) showed a closer relationship to *M. wolinskyi* than to *M. smegmatis*, despite having only an average nucleotide identity (ANI) of about 97.49% compared with *M. wolinskyi*, which is above the cut-off of 95%–96% proposed by Richter & Rossello-Mora to classify bacteria as the same species, but below the cut-off of 98% that is generally used to distinguish between subspecies (Fig. 1) (4–6).

**Fig 2:**
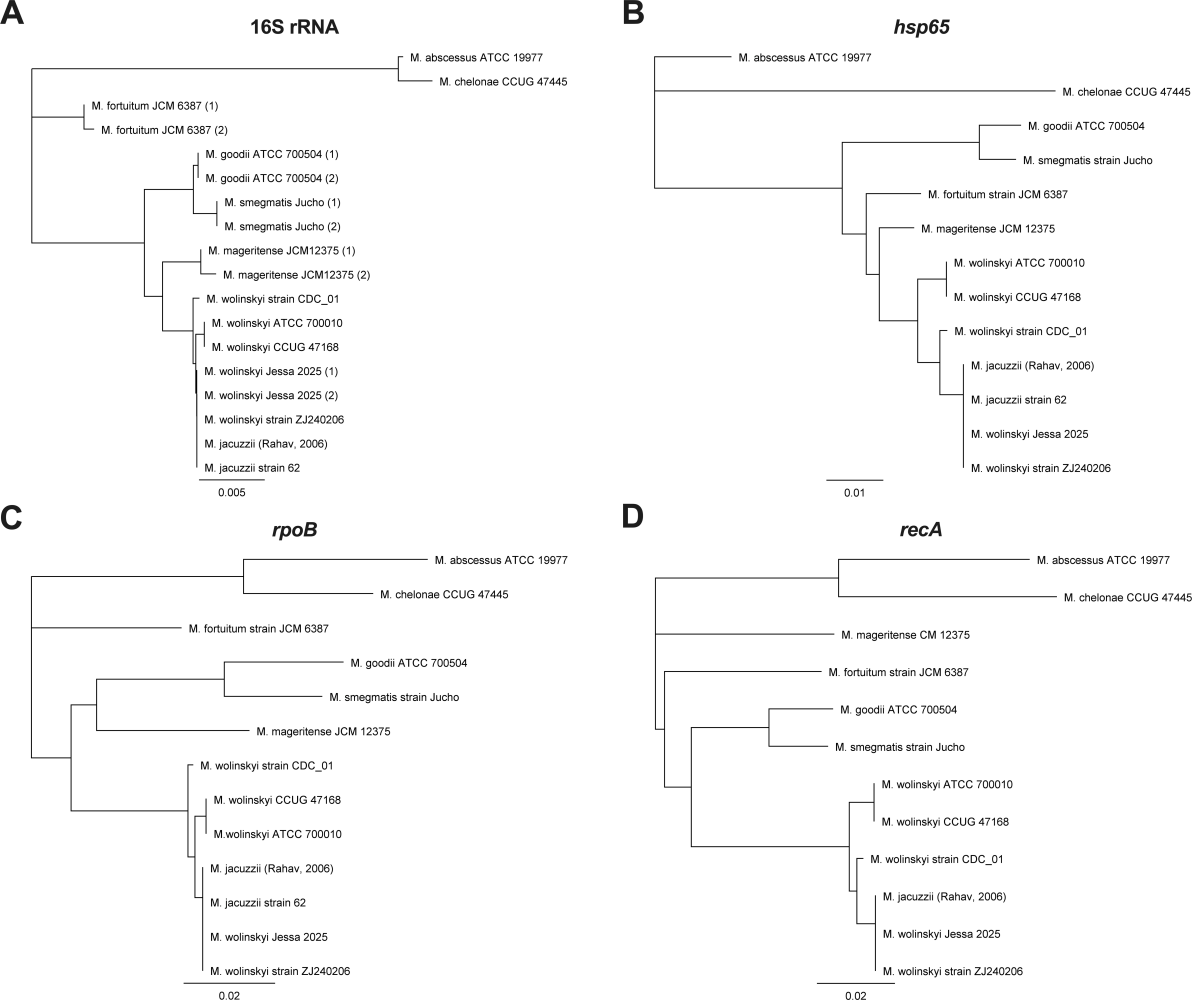
Phylogenetic analysis based on 16S rRNA (**A**), *hsp65* (**B**)*, rpoB* (**C**), and *recA* (**D**) sequences for several fast-growing *Mycobacteria*. For the 16S rRNA gene, the corresponding near full-length nucleotide sequence (1,388 bp) was used, while for the *hsp65* (372 bp)*, rpoB* (723 bp), and *recA* (951 bp) genes, a partial sequence of identical length was used for the phylogenetic analysis, which was performed via the Geneious Tree Builder using the Neighbor-Joining tree build method and the Tamura-Nei Genetic Distance model (Geneious Prime GraphPad software). In cases where a particular strain harbored multiple 16S rRNA copies, these are indicated by (1) and (2).

Because of discrepant 16S rRNA sequencing results and low ANI percentage compared with the *M. wolinskyi* reference genome (strain ATCC 700010), a phylogenetic analysis was performed. Although phylogenetic analysis of the 16S rRNA genes revealed that there was only one SNP difference between the 16S rRNA sequences of the *Mycobacterium* isolate in this study and other *M. wolinskyi* strains*,* the differences between their *hsp65*, *rpoB*, and recA sequences were more pronounced (Fig. 2). The isolate had an identical 16S rRNA, *hsp65*, *rpoB*, and *recA* sequence as the unofficially named *M. jacuzzii* strains described in the studies of Rahav et al. (8) and Sakhaee et al. (9) as well as the *M. wolinskyi* ZJ240206 strain that was isolated from a human host in Beijing in 2024 in contrast to the other *M. wolinskyi* genomes that seemed to be forming separate clusters based on *hsp65*, *rpoB*, and *recA* phylogeny. In addition, the ANI between the *Mycobacterium* isolate in this study and *M. wolinskyi* ZJ240206 was 99.97%, which was considerably higher than the ANI between this isolate and *M. wolinskyi* ATCC 700010 (97.49% as mentioned before), *M. wolinskyi* CCUG47168 (97.48%), and *M. wolinskyi* CDC_01 (97.68%) strains, indicating that the former two could belong to a different subspecies.

The ICD battery and leads were removed, and the pacemaker pocket was debrided. Transesophageal echocardiogram (TEE) after removal showed no residual endocardial vegetations. PET-CT showed no secondary foci of infection but did show FDG-uptake at the pacemaker pocket. Combination therapy of oral moxifloxacin 400 mg once daily and co-trimoxazole 960 mg twice daily was initiated and continued for 6 months. Identification to subspecies level was not required for adequate treatment, since antibiotic resistance differences within the species are not known. Follow-up consisted of monthly transthoracic echocardiogram (TTE), PET-CT, and TEE after 5 months of treatment, which were consistently negative.

## DISCUSSION

Following an outbreak of surgical wound infections in 15 women with breast implants in Israel in 2003, the unofficially described *Mycobacterium jacuzzii* was first reported (8). This *Mycobacterium* was found to be genetically similar to previously described *M. wolinskyi* strains (8). WGS showed 98% identity in the h*sp65* sequence and varying degrees of identity in other genes like *rpoB*, 16S rRNA, *sodA*, and *recA* when compared with other *M. wolinskyi* strains. For instance, the 16S rRNA sequence of *M. jacuzzii* differed by 1 bp from that of *M. wolinskyi* strain ATCC 700010 (8). However, *M. jacuzzii* has not been formally classified as a subspecies of *M. wolinskyi* due to the lack of formal validation under current nomenclature guidelines despite being reported in a case of septic arthritis of the wrist in 2020 (9–11). In 2024*,* this mycobacterium was identified in patients with respiratory failure in China (12).

Similar to *M. wolinskyi,* our strain could show a predilection for device-associated infections, consistent with previous reports involving breast implants. With only four *M. jacuzzi* isolates characterized by WGS, the true prevalence of these strains remains unknown. Given the geographic diversity of cases, it is plausible that our strain is more widespread than currently recognized.

Rapidly growing mycobacteria may be acid-fast variable or negative, especially with fluorochrome stain (13). Negative Ziehl-Neelsen stain in this case highlights the diagnostic challenges associated with certain NTM and the risk of underdiagnosis when relying on diagnostic algorithms based solely on acid-fast staining of positive mycobacterial cultures. While MALDI-TOF MS remains a useful initial identification tool, its limited taxonomic resolution compared with WGS restricts its ability to accurately differentiate between subspecies.

The discrepancy between 1928 Diagnostics and BugSeq results can be explained by inclusion of *M. jacuzzii* in 1928 Diagnostics but not in the BugSeq database. Given minimal differences in the 16S rRNA gene between *M. wolinskyi* and our strain, differentiation by 16S is challenging. As with many NTM isolates, sequencing of genes, such as *hsp65* and *rpoB,* or the entire genome as performed in this study, is needed for accurate identification. The similarity to *M. wolinskyi* ZJ240206 raises questions about subspecies boundaries within the *M. wolinskyi* species and underscores the need for broader phenotypic and genomic studies to improve diagnostic accuracy. The closer phylogenetic relatedness with isolates from Israel, Iran, and China compared with other *M. wolinskyi* strains suggests that this may represent a widely distributed and potentially emerging (sub)clade or even subspecies, but this requires further investigation.
